# Whole-Genome Sequencing-Based Confirmatory Methods on RT-qPCR Results for the Detection of Foodborne Viruses in Frozen Berries

**DOI:** 10.1007/s12560-024-09591-6

**Published:** 2024-04-30

**Authors:** Zhihui Yang, Michael Kulka, Qianru Yang, Efstathia Papafragkou, Christine Yu, Samantha Q. Wales, Diana Ngo, Haifeng Chen

**Affiliations:** Division of Molecular Biology, Office of Applied Research and Safety Assessment, Center for Food Safety and Applied Nutrition, U.S. Food and Drug Administration, 8301 Muirkirk Road, Laurel, MD 20708 USA

**Keywords:** Norovirus, Hepatitis A virus, RT-qPCR, Pre-amplification, Next-generation sequencing

## Abstract

Accurate detection, identification, and subsequent confirmation of pathogens causing foodborne illness are essential for the prevention and investigation of foodborne outbreaks. This is particularly true when the causative agent is an enteric virus that has a very low infectious dose and is likely to be present at or near the limit of detection. In this study, whole-genome sequencing (WGS) was combined with either of two non-targeted pre-amplification methods (SPIA and SISPA) to investigate their utility as a confirmatory method for RT-qPCR positive results of foods contaminated with enteric viruses. Frozen berries (raspberries, strawberries, and blackberries) were chosen as the food matrix of interest due to their association with numerous outbreaks of foodborne illness. The hepatitis A virus (HAV) and human norovirus (HuNoV) were used as the contaminating agents. The non-targeted WGS strategy employed in this study could detect and confirm HuNoV and HAV at genomic copy numbers in the single digit range, and in a few cases, identified viruses present in samples that had been found negative by RT-qPCR analyses. However, some RT-qPCR-positive samples could not be confirmed using the WGS method, and in cases with very high Ct values, only a few viral reads and short sequences were recovered from the samples. WGS techniques show great potential for confirmation and identification of virally contaminated food items. The approaches described here should be further optimized for routine application to confirm the viral contamination in berries.

**Supplementary Information:**

The online version contains supplementary material available at 10.1007/s12560-024-09591-6.

## Introduction

Hepatitis A virus (HAV) and human norovirus (HuNoV) are recognized as major foodborne viral pathogens in the US and worldwide (Scallan et al., 2011). After a long downward trend of HAV infections due to the introduction of HAV vaccines in the 1990s (Werzberger et al., 1992) (Innis et al., 1994), there has recently been a significant increase in US cases due to contaminated imported foods (Center for Disease Control and Prevention) as well as around the world. HuNoV is the leading cause of foodborne outbreaks and is responsible for approximately 58% of cases in the US (Scallan et al., 2011). HuNoV has been estimated to cause approximately 700 million illnesses and 219,000 deaths annually, leading to over $60 billion USD in societal costs worldwide (Bartsch et al., 2016) (Silva et al., 2021). Although close person-to-person contact with an infected person is the major route of transmission, these viruses spread easily and quickly through contaminated surfaces and foods via the fecal–oral route. HAV and HuNoV have very low human infectious doses (HID) and contaminated food items typically carry very few viral particles (Yezli & Otter, 2011) (Bozkurt et al., 2021). These factors pose great challenges for food safety efforts, as levels of detection for current methods are close to or higher than the HID.

Foods such as berries (fresh and frozen) are at particular risk for being contaminated with enteric viruses, partly because the fruits require hand picking and partly because these popular foods are consumed without a kill step; any viruses present from growing, harvesting, packing, and serving will remain capable of causing infections (Maunula et al., 2013; Tavoschi et al., 2015; Torok et al., 2019). More specifically, outbreaks caused by strawberries (Made et al., 2013), raspberries (Sarvikivi et al., 2012) (Saupe et al., 2021), and blackberries (Centers for Disease Control and Prevention) have been recorded worldwide (Bozkurt et al., 2021).

HAV and NoV are small non-enveloped RNA viruses (~ 27–38 nm diameter), with a linear positive-sense single-stranded RNA genome approximately 7.5–7.7 kb in length (Randazzo & Sanchez, 2020) (Chhabra et al., 2019). HAV is a member of the family *Picornaviridae* whose genome contains one open reading frame (ORF) (Costa-Mattioli et al., 2002). The nucleotide variable region located within the viral genome encoding for the VP1/P2A junction has been used for identifying and discriminating between different HAV strains (Brown et al., 1989). Currently, HAV variants infecting humans cluster into genotypes I-III (GI-GIII) and further into subgenotypes GI-A and -B, GII-A and -B, and GIII-A and -B (Singh et al., 2015). Norovirus is a member of the family *Caliciviridae* whose genome contains three ORFs (Cotten et al., 2014). Detection of NoV is typically based on primers/probe which target nucleotide sequences in the ORF1-ORF2 overlapping region of the genome (Cannon et al., 2017). NoV is genetically diverse and phylogenetically clustered into 10 genogroups (GI-GX) and at least 49 genotypes (Chhabra et al., 2019). Strains associated with human illness cluster within GI, GII, GIV, GVIII, and GIX; the most prevalent are GII, particularly strains from genotype GII.4 (Petronella et al., 2018).

RT-qPCR is currently the technique of choice for the detection of HAV or HuNoV extracted from foods in part due to its sensitivity and specificity of target detection. Sanger-based sequencing of amplicons, generated by RT-PCR targeting of variable region(s) of the viral genome, is typically used to provide both confirmation of virus presence and virus genotyping (Coudray-Meunier et al., 2014) (Vinje, 2015). However, testing variables such as the presence of low virus numbers (e.g. typically Ct > 35 for RT-qPCR) alone or in combination with food-derived (e.g. berries) inhibitors of the PCR reaction can negatively impact the quantification and sensitivity, thus overall interpretation, of RT-qPCR results (Steele et al., 2022) (Coudray-Meunier et al., 2014) (Lee et al., 2021). These variables can likewise negatively impact the generation of amplicons for Sanger sequencing. Thus, there is a need for the improvement of current, and/or development of alternative, methods for virus detection, as well as confirmation/identification, particularly for low levels of viral contamination that may be anticipated in food matrices associated with outbreaks of foodborne illness.

Whole-genome sequencing (WGS) is an emerging tool in basic molecular and cell biology research and is now being applied to viral analyses of clinical samples (Houldcroft et al., 2017) as well as research investigation of the utility and application for post-detection, identification, and discrimination of foodborne virus strains associated with outbreaks (Desdouits et al., 2020). WGS has been proposed as an additional approach for investigating foodborne virus transmission and source attribution (Raymond et al., 2022), and it could obtain the whole profile of the full-length viral genome, identify emerging genotypes, and determine variants down to a single nucleotide (Yang et al., 2018). However, unlike clinical samples that contain high levels of virus, environmental and food samples typically contain many fewer viral particles per unit mass or volume of sample. To address the need to sequence the low quantity of HuNoV targets, some researchers use a targeted pre-amplification approach to sequence either the ORF2 and ORF3 (Raymond et al., 2022) regions or the full-length genome (Tohma et al., 2021) (Parra et al., 2017). Yang et al*.* have used a non-targeted pre-amplification approach to perform WGS for HAV and HuNoV present in clinical, culture, and food samples and at varying concentrations (Silva et al., 2021) (Yang et al., 2018) (Yang et al., 2017). Chen et al*.* has applied a non-targeted pre-amplification in combination with WGS in order to detect HuNoV from fecal specimens and HAV from frozen raspberries (Chen et al., 2018) (Chen et al., 2019). Strubbia et al*.* demonstrated that virus sequence detection and identification can be improved by using a virus capture approach (Strubbia et al., 2019). While advances in the technical aspects of WGS have been made, research continues toward establishing whether it may be better to take a metagenomic approach for the identification and confirmation of viruses isolated from foods and water.

**Fig. 1:**
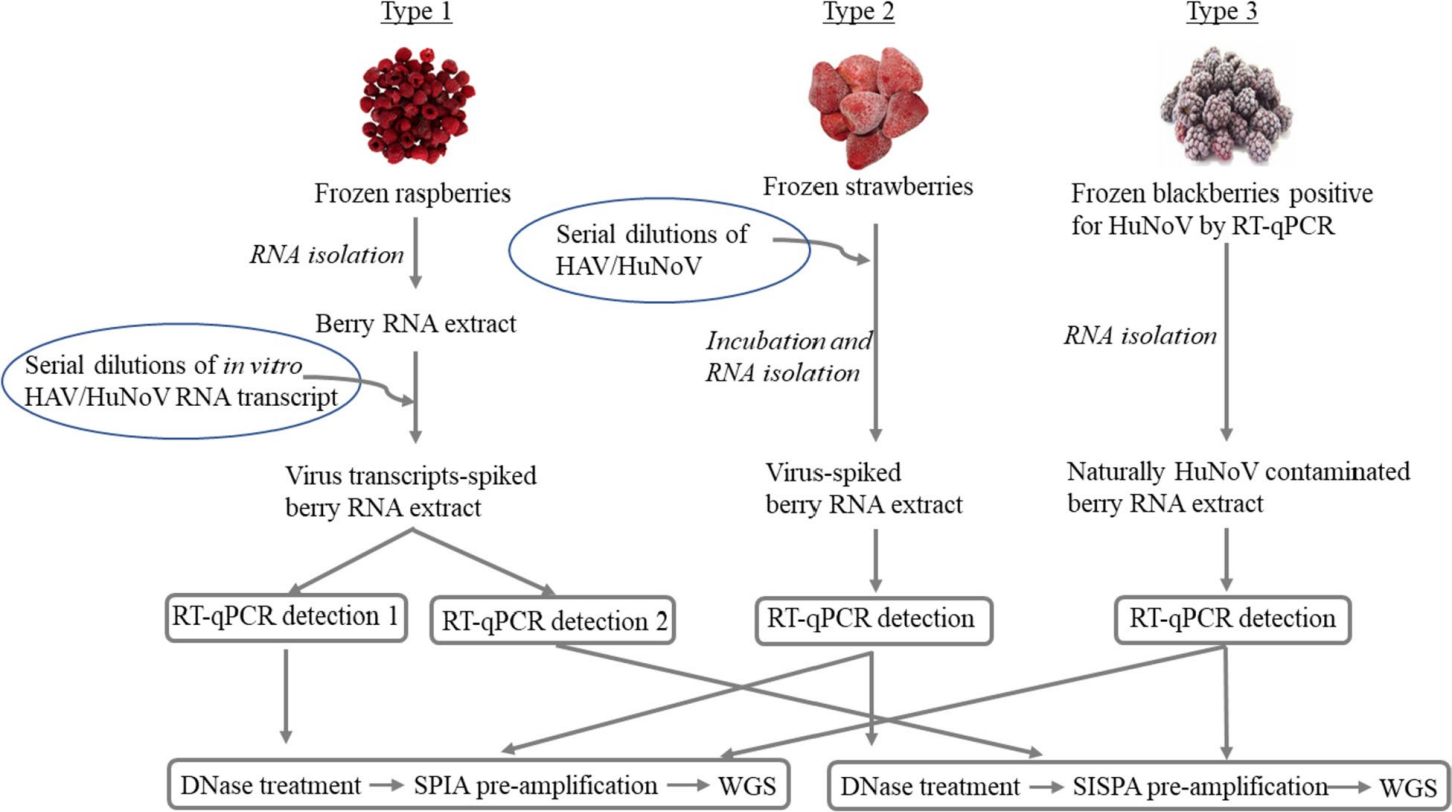
Schematic representation of experimental approach for sample preparation, RNA isolation, PCR detection, and whole-genome sequencing. As described in M&M, sample preparation and RT-qPCR were carried out following BAM protocol except for the exclusion of process control MNV (murine norovirus). Three types of viral samples were employed in this study: (1) serial dilutions of either HAV or HuNoV transcripts were added into the total RNA isolated from frozen raspberries; (2) serial dilutions of either HAV or HuNoV were seeded into 50 g frozen strawberries, RNA isolation was performed after incubation at room temperature for at least one hour; (3) RNA was isolated from frozen blackberries naturally contaminated with norovirus. RT-qPCR was conducted with each RNA sample, followed by both SPIA-WGS and SISPA-WGS

The goal of the current investigation was to improve upon earlier strategies through the combination of WGS with either of two non-targeted pre-amplification methods (SPIA—Single-Primer Isothermal Amplification; SISPA—Sequence-Independent, Single-Primer Amplification). Both pre-amplification methods were assessed using three types of frozen berry samples (Fig. 1). Type 1 samples had full-length in vitro transcribed viral RNA transcripts added into raspberry RNA extracts, Type 2 samples had virus spiked onto strawberries, and Type 3 samples were blackberries naturally contaminated with HuNoV (post-investigational samples from an FDA field lab). Using these sample types, we investigated if this sequencing approach could be potentially used as a confirmatory method for RT-qPCR virus-positive berry samples, especially for samples with high Ct values, and further genotyping if sufficient sequences can be obtained.

## Materials and Methods

### In Vitro* Viral RNA Transcripts*

The RNA expression vectors pHAV/7.1 and pHuNoV/MD145 were used for in vitro transcription of RNAs, each encoding one complete genome sequence for either HAV or HuNoV GII.4, respectively (Yang et al., 2017; Yu et al., 2016). The RNAs were produced from in vitro transcription cassettes, using a commercial kit (Megascript, Ambion, Inc.) designed for SP6 (HAV7.1) and T7 (HuNoV GII.4)-driven transcription, according to the manufacturer’s instructions. Following enrichment/purification (Poly(A) Purist Kit, Ambion), RNA pA + transcripts were concentrated by ammonium acetate precipitation at − 20 °C, centrifugation, and resuspension of the RNA pellets in RNA Storage solution (Ambion).

### Virus Stocks

Hepatitis A virus HM175/18f, purchased from ATCC (catalog number: VR-1402), propagated and purified as previously described (Kulka et al., 2009) (Dotzauer et al., 2000), was used to artificially contaminate the strawberries. The viral titer of this stock was determined to be 3 × 10^8^ PFU/mL by plaque assay (Hida et al., 2013) on fetal rhesus monkey kidney cells (FRhK4, obtained from Dr. G. Kaplan, CBER, FDA) and further determined to be 4.1 × 10^10^ cps copies (cps)/µL by RT-qPCR assay. The HuNoV GII strain (GenBank accession number MK301293) (Yang & Mammel, 2019) used to artificially contaminate the strawberries was associated with a sporadic case of acute gastroenteritis in Maryland in 2018. It was prepared as a 10% (wt/vol) fecal suspension in phosphate-buffered saline. RNA was extracted using a QIAamp viral RNA mini kit (Qiagen). As measured by RT-qPCR, the viral load of this fecal suspension was approximately 3 × 10^6^ cps copies (cps)/µL. Both HAV and HuNoV were aliquoted separately and stored at − 80 °C.

### Berry Source, Preparation of Berry Concentrates, and Isolation of Viral RNA from Berry Concentrates

Frozen raspberries and frozen strawberries were purchased from a local retail store and stored at − 20 °C until use. Naturally contaminated blackberries, which had been previously tested positive for HuNoV GII by RT-qPCR, were received as 3 opened 1-pound bags (designated as bags A, B, and C). Four 50 g samples were taken from each of the three bags (samples designated as A-1 to A-4, B-1 to B-4, and C-1 to C-4).

Frozen raspberries, virus-spiked frozen strawberries, and naturally contaminated blackberry sample concentrates were prepared using the FDA Bacteriological Analytical Manual (BAM) protocol for Concentration and Extraction, of Enteric Viruses from Soft Fruit, with the exception that the process control virus was not added (FDA, 2022). Briefly, 30 mL of Glycine Beef Extract Buffer (0.1 M Tris, 0.05 M glycine, and 6% beef extract, pH 9.5) and 50 µL of pectinase were added to 50 g of berries in a Whirl–Pak filter bag (Nasco, Fort Atkinson, WI). The bag was shaken at 150 rpm for 15 min at room temperature. Then, the eluate was collected and clarified by centrifugation at 12,000 × *g* for 15 min at 4 °C. The supernatant was recovered and transferred to ultracentrifuge tubes. Virus was pelleted at 170,000 × g for 45 min at 4 °C and resuspended in 600 µL of PBS, followed by chloroform extraction. Concentrates were stored at -20 °C until further processing.

Following the BAM protocol (FDA, 2022), viral RNA was isolated from each of the berry concentrates. Briefly, 200 µL of berry concentrate was lysed with 560 µL of Buffer AVL without carrier RNA. After incubation with 100 µL 2 M Potassium Acetate solution on ice for 15 min, the lysate was clarified by centrifugation at 16,000 × g for 10 min before passing through a QIAshredder column. The supernatant of the flow-through was used for RNA extraction with QIAamp Viral RNA mini kit. RNA was eluted in 100 µL Buffer AVE and further cleaned by two One-Step PCR Inhibitor Removal columns (Zymo Research). Purified RNA was stored at -80 °C until use.

### Sample Types

#### Type 1 Samples: Viral Transcripts in RNA Extracts Derived from Frozen Raspberries

Type 1 samples used RNA extracts from frozen raspberries containing either HAV or HuNoV RNA transcript. Total RNA isolated from the raspberry concentrates was used as the diluent to generate serial tenfold dilutions, in quintuplicate, of either HAV or HuNoV RNA transcripts ranging from 10^5^ to 10^–1^ RNA cps per 3 µL of raspberry extract. This dilution series was aliquoted to give multiple identical dilution series sets that were stored at − 20 °C. For each sequencing experiment, a separate dilution series was thawed, and RT-qPCR performed on each dilution followed by sequencing.

#### Type 2 Samples: Virus Spiked onto Frozen Strawberries

Type 2 samples used frozen strawberries spiked with 100 µL of either HAV or HuNoV. The frozen strawberries were thawed at room temperature, then 50 g/sample was weighed, sliced, and then virus added. Spiking levels per sample ranged between approximately 10^7^–10^2^ PFU for HAV and 10^8^–10^2^ RNA cps for HuNoV GII. Samples were left to air dry for at least one hour at room temperature, up to overnight at 4 °C. Each spiked sample was then transferred to a filtered Whirl–Pak® plastic bag (ThermoFisher Scientific) and processed as described in Sect. "Berry source, preparation of berry concentrates, and isolation of viral RNA from berry concentrates". All experiments were carried out in 3 or 4 replicates.

#### Type 3 Samples: Naturally Contaminated Frozen Blackberries

The source of Type 3 samples was frozen blackberries naturally contaminated by norovirus. Three one-pound bags of frozen blackberries (Type 3 sample) were previously detected as HuNoV GII positive by RT-qPCR, with Ct values reported as 46.5/43.4/undetermined for 3 replicates by the provider (FDA, post-investigation sample). Four samples of 50 g each were taken from each bag, 500 µL of berry concentrate was obtained from each sampling and 140 µL of each blackberry concentrate from each sample was used for RNA isolation followed by RT-qPCR testing. In addition, the remaining concentrates of each sample from the same bag [(500–140) µL/sampling × 4 samplings/bag = 1440 µL] were pooled and concentrated using Amicon Ultra-2 ml Centrifugal Filters (Regenerated Cellulose 100 k NMWL, Millipore Sigma). The recovered volume of each concentrated berry concentrate was 82, 91 and 90 µL for bags A, B, and C, respectively. Preparation of berry concentrates and isolation of viral RNA were performed following the BAM protocol as described in Sect. "Berry source, preparation of berry concentrates, and isolation of viral RNA from berry concentrates".

### Viral Detection by Real-Time RT-PCR

Following the BAM protocol (FDA, 2022), RNA isolated from all three sample types was quantified using two multi-lab validated TaqMan RT-qPCR assays (one for HAV RNA and one for HuNoV GII RNA), run on an ABI 7500 Fast system (Life Technologies). As a standard method, all the information required to perform the HAV and NoV GII RT-qPCR is detailed in the BAM’s official website, including required reagents and equipment, sample preparation, primers/probes’ sequences, reaction mix setup, cycle condition, and data analysis. The RT-qPCR reactions were completed in a 96-well format. Each well contained the following: (1) 3 µL of raspberry RNA containing viral RNA transcript (Type 1 samples), or 3 µL of RNA from virus-spiked strawberries (Type 2 samples), or 3 µL of RNA from naturally contaminated blackberry samples (Type 3 samples); and (2) 0.2 µL of an Internal Amplification Control (IAC) RNA. All RNA samples as well as reaction controls and serial dilutions of either HAV or HuNoV transcript (10^5^–10^1^ cps/rxn) to generate the standard curve were run in triplicate.

### Grouping RT-qPCR Results by Ct Values

We grouped the results of each assay based on the RT-qPCR data: (1) Group I samples that had Ct values less than or equal to 35 (rarely reported for the contaminated food items); (2) Group II samples that had Ct values between 35 and 40 (often reported for the contaminated food items, especially values at the high end of 30 s); and (3) Group III samples that had Ct values equal to or higher than 40 (the most frequently reported for the contaminated food items and are the most challenging for determining true or false positivity).

### Viral Detection by Whole-Genome Sequencing

#### DNase Treatment

In preparation for the SPIA or SISPA pre-amplification strategies, each RNA sample was subjected to DNase I (Qiagen) digestion. Fifteen µL of RNA was incubated with DNase I at 25 °C for 10 min followed by RNA purification using RNeasy MinElute Cleanup kit (Qiagen) according to the manufacturer’s instructions. Each purified RNA sample was then divided for pre-amplification by either SPIA or SISPA.

#### Pre-Amplification with SPIA Technology

For single primer isothermal amplification (SPIA), purified DNase I-treated RNA samples of each type were reverse-transcribed and amplified using the Ovation RNA Sequencing (RNA-Seq) System Version 2 Kit (NuGen), according to the manufacturer’s protocols (Chen et al., 2018) (Chen et al., 2019). Briefly, 5 μL of sample RNA was subjected to first-strand cDNA synthesis using a combination of random hexamers and poly-T RNA/DNA chimeric primer mix at 65 °C for 5 min, then at 4 °C for 1 min, 25 °C for 10 min, 42 °C for 10 min, and 70 °C for 15 min. The second cDNA strand was generated in the presence of RNA-dependent DNA polymerase at 4 °C for 1 min, 25 °C for 10 min, 50 °C for 30 min, and 80 °C for 20 min. The resulting double-stranded cDNA was cleaned using RNA Clean XP purification beads (Beckman Coulter) and then amplified on beads using SPIA. The SPIA reaction was performed at 4 °C for 1 min, 47 °C for 60 min, and 80 °C for 20 min and hold at 4 °C. After removing the beads, 40 μL of amplified dsDNA was purified using Qiagen MinElute Reaction Cleanup Kit (Qiagen) and eluted in 30 μL of buffer. The yield of these amplified products was measured using a Qubit 3 fluorometer (Invitrogen) prior to further sequencing use.

#### Pre-Amplification with SISPA Technology

The purified DNase-treated RNA of each sample type was pre-amplified using the SISPA technique, following the protocol described by (Kapusinszky et al., 2017; Victoria et al., 2008). Reverse transcription was performed by incubating 10 µL of RNA and a random primer with a fixed 18-nt at the 5’ end (5’-GCCGACTAATGCGTAGTCNNNNNNNNN-3’) at 85 °C for 2 min. The first strand of cDNA was synthesized by incubating the mixture of primed RNA, SuperScript III reverse transcriptase (Invitrogen), dNTPs (deoxynucleotide triphosphates), DTT (dithiothreitol), and 5 × first-strand buffer (Invitrogen) together at 25 °C for 10 min, followed by 50 °C for 1 h. Double-stranded cDNA was synthesized with the Klenow fragment enzyme (New England Biolabs) using a temperature cycling pattern of 95 °C for 2 min, 4 °C for 2 min, and 37 °C for one hour. To amplify this double-stranded cDNA by PCR, it was incubated with the random primer above without Ns (5’-GCCGACTAATGCGTAGTC-3’), dNTPs, and AmpliTaq Gold DNA polymerase (Life Technologies) at 95 °C for 5 min, 30 cycles of 95 °C for 30 s, 55 °C for 30 s, and 72 °C for 2.5 min, followed by the final extension at 72 °C for 10 min. The resulting PCR products were purified and size-selected using Agencourt AMPure XP beads (Beckman Coulter).

#### WGS Library Preparation, Sequencing, and Data Analysis

To create WGS libraries from products of the SPIA or SISPA amplification, we used the Nextera XT DNA Sample Preparation Kit (Illumina), following the manufacturer’s protocol. No more than 12 libraries were pooled on each run, which were sequenced on the MiSeq Platform (Illumina) with MiSeq Reagent Kit v2 to generate paired-end reads of 250 bp in length. Raw sequencing data were imported into CLC Genomics Workbench (CLC GWB-version 19, Qiagen) and analyzed with default parameters, unless otherwise specified.

Following both adapter- and quality-trimming (quality score limit = 0.05, maximum number of ambiguities = 2), we performed sequence-based alignment against our local database including the representative full-length norovirus sequences downloaded from NCBI (Yang et al., 2017). The top matched sequence was used as the reference sequence, and then, we performed reference-based alignment on the reads from each sample. Finally, the viral sequence obtained from each sample was genotyped using the Norovirus Genotyping Tool Version 2.0 (Kroneman et al., 2011), the CaliciNet Human Calicivirus Typing Tool (https://norovirus.ng.philab.cdc.gov), or Hepatitis A Virus Genotyping Tool Version 1.0 (https://www.rivm.nl/mpf/typingtool/hav/).

## Results

This study used SPIA and SISPA pre-amplification in conjunction with WGS toward improving sequencing sensitivity of HAV and HuNoV extracted from three types of berry samples (Fig. 1). Prior to WGS, RT-qPCR was carried out for each RNA sample, and the results were grouped based on Ct values as described in Sect. "Grouping RT-qPCR results by Ct values".

### WGS Analysis of Type 1 Samples: Viral RNA Transcripts Spiked into Frozen Raspberry RNA Extracts

#### WGS Results of Type 1 Samples Containing HAV RNA Transcripts

The RT-qPCR results showed the Ct values were between 28.2 and 31.9 for the highest concentration of HAV RNA transcript 10^5^ cps/3 µL, and above 41.0 or undetermined for the lowest concentration 0.1cps/3 µL (Supplementary Table 1a and 1b). Five sequencing runs were performed for WGS with SPIA pre-amplification (SPIA-WGS), and four performed for WGS with SISPA pre-amplification (SISPA-WGS). The WGS results were summarized to assess the overall performance of each WGS (Supplementary Table 1a and 1b).

##### Results of SPIA-WGS on HAV RNA Transcript in Raspberry RNA Extracts

WGS with SPIA pre-amplification was carried out on 5 sets of raspberry extracts spiked with 7 serial dilutions of HAV RNA transcript. In total, 27 PCR-positive samples were sequenced and 23 (85%) of them were confirmed as containing HAV through this SPIA-WGS strategy. For the 9 samples in Group I, for which the corresponding input was equal to or greater than 1,000 cps/3 µL HAV RNA, total reads ranged from 0.04 to 4.04 M and the percentage of HAV reads ranged from 7.2 × 10^–4^ to 3.8 × 10^–1^. Nearly full-length HAV transcript sequences (6,612 to 7,423 bp) were generated from these 9 samples and correctly typed as HAV IB. (See Table 1, and Supplemental Table 1a). For the 5 samples in Group II (100–1,000 cps/3 µL HAV RNA), total reads ranged from 0.08 to 5.10 M, the percentage of HAV reads ranged from 1.9 × 10^–4^ to 3.9 × 10^–3^ and 3,258 to 7,417 bp in length. All samples were correctly genotyped as HAV IB (Table 1 and Supplementary Table 1a). Of the 13 Group III samples (equal to or less than 10 cps/3 µL HAV RNA), 10 samples yielded 0.05–7.04 M total reads, of which ≤ 4.3 × 10^–4^% were HAV reads. The recovered HAV sequences varied widely in length from 31 bp to nearly full length (7,268 bp), 9 of these 10 samples were genotyped as HAV IB, and the other one with the shortest sequence of 31 bp was not assigned by the HAV Genotyping Tool. HAV could not be detected in the 3 other Group III samples. Interestingly, HAV sequences ranging from 387 to 7,139 bp were recovered from 4 out of five PCR-negative (Ct undetermined) samples and were identified as HAV at either a genotype or species level (Supplemental Table 1a).

**Table 1:** Summary of the sequencing results of HAV transcript-added RNA extracts from frozen raspberries

Ct value (Ct Group)	Copies/3 uL		Total reads (M)		% of HAV reads		Length of HAV fragment by WGS (bp)		Identification (Confirmation rate; N) *	
	SPIA	SISPA	SPIA	SISPA	SPIA	SISPA	SPIA	SISPA	SPIA	SISPA
Ct ≤ 35 (Group I)	1E3 -1E5	1E4 -1E5	0.04–4.04	3.68–6.71	7.2E-4–3.8E-1	2.1E-5–6.0E-1	6612–7423	5286–7439	9-GT	7–GT
									(100%; N = 9)	(100%; N = 7)
Ct ≥ 35 and ≤ 40 (Group II)	1E2 -1E3	1E2 -1E3	0.08–5.10	3.63–6.57	1.9E-4–3.9E-3	4.1E-6–1.2E-2	3258–7417	1149–7326	5-GT	6-GT; 1-SPP
									(100%; N = 5)	(100%; N = 7)
Ct ≥ 40 (Group III)	< 1E2	< 1E2	0.05–7.04	3.91–6.98	0 .0–7.5E-4	8.5E-7–1.68E-4	0–7268	272–7166	9-GT; 4-Und	9-GT
									(69%; N = 13)	(100%; N = 9)

^*^GT genotype, *SPP* species, *Und*. Undetectable, *N* sample number, *Confirmation rate* Percentage of RT-qPCR-positive samples confirmed by WGS

##### Results of SISPA-WGS on HAV RNA Transcript in Raspberry RNA Extracts

WGS with SISPA pre-amplification was carried out on 4 sets of spiked berry RNA extracts. Group I contained 7 samples with total reads ranging from 3.68 to 6.71 M, and the percentage of HAV reads ranging from 2.1 × 10^–5^ to 6.0 × 10^–1^, with 7 out of 7 (100%) samples correctly genotyped as HAV IB (Table 1 and Supplementary Table 1b). Assembled sequences ranged from 5,286 to 7,439 bp representing nearly full-length HAV sequences. Among the 7 samples in Group II, total reads ranged from 3.63 to 6.57 M and the percentage of HAV reads ranged from 4.1 × 10^–6^ to 1.2 × 10^–2^. Six of the 7 Group II samples were correctly genotyped as HAV IB, with HAV sequences ranging from 1,767 to 7,326 bp. The remaining Group II sample had a Ct value of 39.6 with a sequence of 1,149 bp that could be genotyped at the species level. Nine samples were in Group III, from which we obtained 3.91 to 6.98 M total reads and HAV reads at 1.7 × 10^–4^ percent or below. All 9 samples in this group (100%) could also be genotyped as HAV IB, even though the lengths of recovered HAV sequences differed widely from 272 bp to nearly full length at 7,166 bp.

In total, 23 RT-qPCR-positive samples were sequenced and 100% confirmed as HAV with SISPA-WGS, of which 22 samples were correctly identified at genotype level and the other one at species level. Interestingly, one out of 2 samples not detected by RT-qPCR was WGS positive, with a sufficient 581 bp sequence recovered to be identified as HAV IB. (Supplementary Table 1b). The possibility of significant RT-PCR inhibition was ruled out (based on the BAM guidelines) since the average of the IAC Ct values for the sample replicates was less than 4.0 Ct’s greater than the Negative Control IAC Ct value.

#### WGS Results of Type 1 Frozen Raspberry RNA Samples Containing HuNoV RNA Transcripts

The RT-qPCR results showed the Ct values were between 25.6 and 27.3 for the highest concentration of HuNoV RNA transcript (10^5^ cps/3 µL) and were above 42.0 or undetermined for the lowest concentration (0.1cps/3 µL) (Supplementary Table 1c and 1d). Three sequencing runs were performed for WGS with SPIA pre-amplification (SPIA-WGS), and three performed for WGS with SISPA pre-amplification (SISPA-WGS). The WGS results were summarized to assess the overall performance of each WGS (Supplementary Table 1c and 1d).

##### Results of SPIA-WGS on HuNoV RNA Transcripts in Raspberry RNA Extracts

In total, 20 PCR-positive samples were sequenced, and all (100%) could be confirmed using our SPIA-WGS strategy. For the 9 samples in Group I (corresponding input was 1000 cps/3 µL or more total RNA), total reads ranged from 0.04 to 5.02 M and the percentage of HuNoV reads ranged from 3.5 × 10^–5^ to 5.4 × 10^–3^. We recovered HuNoV transcript sequences (2,394–7,556 bp) from 9 out of 9 (100%) from these samples, which were detected and identified as HuNoV GII.4[P4] at genotype level (Table 2 and Supplementary Table 1c). For the six samples in Group II (corresponding input of 100–1000 cps/3 µL total RNA), total reads ranged from 0.01 to 5.58 M of which the percentage of HuNoV reads ranged from 4.8 × 10^–6^ to 7.6 × 10^–4^. The recovered HuNoV sequences from Group II samples ranged from 920 to 4,348 bp. Four of these six samples could be identified and genotyped as HuNoV GII.4[P4], the other two were assigned at HuNoV species level. The five samples in Group III provided 0.02–4.70 M of total reads (input of 10 cps/3uL or less total RNA), of which the percentage of HuNoV reads was 1.1 × 10^–6^ or less. Recovered HuNoV sequences from these last 5 samples varied widely in length, from 412 to 1,745 bp. Only one Group III sample could be genotyped as HuNoV GII.4[P4], three as HuNoV at the species level, and one was undetectable. One PCR-negative sample was also sequenced, and no norovirus reads were generated (Supplementary Table 1c).

**Table 2:** Summary of the sequencing results of norovirus transcript-added RNA extracts from frozen raspberries

Ct value (Ct Group)	Copies/3 uL		Total reads (M)		% of HuNoV reads		Length of HuNoV fragment by WGS (bp)		Identification (Confirmation rate; N) *	
	SPIA	SISPA	SPIA	SISPA	SPIA	SISPA	SPIA	SISPA	SPIA	SISPA
Ct ≤ 35 (Group I)	1E3 -1E5	1E3 -1E5	0.04–5.02	3.67–5.44	3.5E-5–5.4E-3	0.0E0–5.0E-4	2394–7556	0–7569	9-GT (100%; N = 9)	7-GT; 1-SPP; 1-Und (89%; N = 9)
Ct ≥ 35 and ≤ 40 (Group II)	10–100	10–100	0.01–5.58	3.92–5.04	4.8E-6–7.6E-4	0.0E0–4.4E-6	920–4348	0–2170	4-GT; 2-SPP (100%; N = 6)	1-GT; 2-SPP; 2-Und (60%; N = 5)
Ct ≥ 40 (Group III)	< 10	< 10	0.02–4.70	4.05–6.21	1.1E-6–4.9E-4	0.0E0–4.8E-7	412–1745	0—225	1-GT; 4-SPP (100%; N = 5)	2-SPP; 3-Und (40%; N = 5)

^*^GT genotype, *SPP* species, *Und.* Undetectable, *N* sample number, *Confirmation rate* Percentage of RT-qPCR-positive samples confirmed by WGS

##### Results of SISPA-WGS on HuNoV RNA Transcripts in Raspberry RNA Extracts

Among the 19 RT-qPCR-positive samples, 13 (68%) of them were confirmed by our SISPA-WGS strategy. For the nine samples in Group I (corresponding input equal to or greater than 1000 cps/3 µL total RNA), the total reads ranged from 3.67 to 5.44 M, of which the percentage of HuNoV reads ranged from 0 to 5.0 × 10^–4^, and the recovered HuNoV transcript sequences ranged from 0 to 7,569 bp in length. Of these 9 samples, 7 were detected and identified as HuNoV GII.4[P4] at genotype level, 1 sample could be placed at species level and the last sample was undetectable (Table 2 and Supplementary Table 1d). For the 5 samples in Group II (corresponding input of 100–1,000 cps/3 µL total RNA), the total reads ranged from 3.92 to 5.04 M, the percentage of HuNoV reads ranged from 0 to 4.4 × 10^–6^, and the recovered HuNoV sequences ranged from 0 to 2,170 bp in length. Among those samples, one was identified and genotyped as HuNoV GII.4[P4], two were detected at HuNoV species level and 2 were undetectable. From the final five samples in Group III, which had a Ct value above 40 (input equal to or less than 10 cps/3 µL total RNA), 4.05 to 6.21 M total reads were obtained, of which 4.8 × 10^–7^% or less were HuNoV reads. Those recovered HuNoV sequences ranged from 0 to 225 bp. Two out of 5 samples were detected as HuNoV at species level, and 3 were undetectable. Two PCR-negative samples were also sequenced, and no norovirus was detected (Supplementary Table 1d).

### WGS Analysis of Type 2 Samples: Viruses Spiked onto Frozen Strawberries

#### WGS Results of HAV-Spiked Type 2 Samples

Frozen strawberries spiked with a serial dilution of HAV HM175/18f were extracted for RNA (Fig. 1). RT-qPCR was carried out and the results showed the Ct values for the highest concentration of undiluted virus (3 × 10^6^ cps/µL) were between 30.0 and 30.5, and the lowest concentration using a 10^5^ dilution (3 × 10^1^ cps/µL) was above 38.9 or undetermined (Supplementary Table 1e and 1f). The WGS results were summarized to assess the overall performance of each WGS (Supplementary Table 1e and 1f).

##### Results of SPIA-WGS on HAV Spiked onto Frozen Strawberries

WGS with SPIA pre-amplification was carried out on 3 sets of HAV-spiked berry samples. In total, 14 PCR-positive samples were sequenced of which 13 (92.9%) of them were confirmed by our SIPA-WGS strategy. Our analysis of five samples in Group I obtained total reads ranging from 0.23 to 8.43 M, and the percentage of HAV reads was 1.4 × 10^–4^ or below. The HAV sequences recovered (1925 to 6919 bp in length) allowed 5 out of 5 samples (100%) to be detected and identified as HAV IB at genotype level (Table 3 and Supplementary Table 1e). For the 8 samples in Group II (corresponding input level ranged from 101 to 792 cps/µL RNA), total reads ranged from 0.26 to 5.22 M and the percentage of HAV reads ranged from 1.9 × 10^–6^ to 3.3 × 10^–5^. Seven out of these 8 samples (87.5%), from which we recovered HAV sequences ranging from 141 to 2136 bp, were identified and genotyped as HAV IB. One sample could be detected as HAV but not genotyped. The remaining sample in Group III, which had a Ct greater than 40 and total reads of 0.22 M, could not be detected using SPIA-WGS. One PCR-negative sample was also sequenced and no HAV was detected (Supplementary Table 1e).

**Table 3:** Summary of the sequencing results of HAV-spiked frozen strawberry samples

Ct value (Ct Group)	Cps for WGS*	Total reads (M)		% of HAV reads		Length of HAV fragment by NGS (bp)		Identification (Confirmation rate; N) **	
SPIA/SISPA		SPIA	SISPA	SPIA	SISPA	SPIA	SISPA	SPIA	SISPA
Ct ≤ 35 (Group I)	4247–19,507	0.23–8.43	4.41–5.81	3.6E-5–1.4E-4	9.1E-7–2.8E-4	1925–6919	539–5671	5-GT	4–GT
								(100%; N = 5)	(100%; N = 4)
Ct ≥ 35 and ≤ 40 (Group II)	101–792	0.26–5.22	3.20–5.76	1.9E-6–3.3E-5	0–3.8E-6	141–2136	0–790	7-GT; 1-SPP	6-GT; 2-Und
								(100%; N = 8)	(75%; N = 8)
Ct ≥ 40 (Group III)	6.6	0.22	5.83	0	6.9E-7	0	748	1-Und	1-GT
								(0%; N = 1)	(100%; N = 1)

^*^Cps total copy numbers of HAV input for WGS

^**^HAV hepatitis A virus, *GT* genotype, HAV 1B, *SPP* species, *Und*. Undetectable, *N* sample number, *Confirmation rate* Percentage of RT-qPCR-positive samples confirmed by WGS

##### Results of SISPA-WGS on Frozen Strawberries Spiked with HAV

Virus-spiked strawberries were also subjected to WGS with SISPA pre-amplification. In total, 13 PCR-positive samples from 3 dilution sets were sequenced; 11 (84.6%) of these were confirmed by the SISPA-WGS strategy. Specifically, four samples in Group I obtained total reads ranging from 4.41 to 5.81 M, and the percentage of HAV reads was at or below 2.8 × 10^–4^. Recovered HAV sequences ranged from 539 to 5,671 bp in length, and all Group I samples (100%) were detected and identified as HAV IB at genotype level (Table 3 and Supplementary Table 1f). For the 8 samples in Group II, total reads ranged from 3.20 to 5.76 M, the percentage of HAV reads was at or below 3.8 × 10^–6^, and recovered HAV sequences were between 0 and 790 bp. Among Group II samples, 6 out of 8 could be identified and genotyped as HAV IB; two were undetectable. Group III contained one sample with Ct greater than 40 and was detected and identified as HAV IB from the 748 bp recovered HAV sequence. One PCR-negative sample was also sequenced and no HAV was detected (Supplementary Table 1f).

#### WGS Results from Frozen Strawberry Samples Spiked with HuNoV

Frozen strawberries spiked with a serial dilution of a norovirus GII.4 strain were extracted for RNA (Fig. 1). The RT-qPCR results showed the Ct values were between 26.7 and 27.5 for the highest concentration of undiluted stool stock, greater than 41.2 or undetermined for the lowest concentration 10^5^ diluted virus or 3 × 10^1^ cps/µL (Supplementary Table 1 g and 1 h). Then the RNA samples were used for SPIA-WGS and SISPA-WGS. The WGS results were summarized to assess the overall performance of each WGS (Supplementary Table 1 g and 1 h).

##### Results of SPIA-WGS on Frozen Strawberries Spiked with HuNoV

SPIA pre-amplification followed by WGS was carried out on 4 sets of HuNoV-spiked berry samples. In total, 24 PCR-positive samples were sequenced and 18 (75%) of them were confirmed by the SIPA-WGS strategy. Eleven samples in Group I (corresponding to 73–8,911 cps of viral genome per µL RNA) obtained total reads ranging from 0.42 to 9.05 M, of which the percentage of HuNoV reads was between 2.0 × 10^–3^ and 4.3 × 10^–6^. The recovered HuNoV sequences were between 755 and 7,023 bp in length, allowing 10 out of these 11 samples to correctly identify and typed as HuNoV GII.6[P7]. The last sample in this set could only be identified as HuNoV at the species level (Table 4 and Supplementary Table 1 g). For the 9 samples in Group II (corresponding to 1.2–30 cps/µL RNA), the total reads ranged from 0.13 to 26.1 M and the percentage of HuNoV reads was 8.3 × 10^–7^ or below. Six out of these 9 samples, from which we recovered HuNoV sequences up to 3,024 bp, were identified as HuNoV at the species level only. HuNoV could not be detected in the other 3 samples in that group. The last 4 samples in Group III (corresponding to 0.8 cps/µL RNA or less) obtained 2.6 × 10^–6^ or less percentages of HuNoV reads, resulted in recovery of 808 bp or less of HuNoV sequences. In one of these samples with high Ct value, we could detect and identify HuNoV at the norovirus species level by our SPIA-WGS strategy, but no virus could be detected in the remaining 3 samples.

**Table 4:** Summary of the sequencing results of HuNoV-spiked frozen strawberry samples

Ct value (Ct Group)	Cps for WGS*	Total reads (M)		% of HuNoV reads		Length of HuNoV fragment by WGS (bp)		Identification (N / Confirmation rate; N) **	
SPIA/SISPA		SPIA	SISPA	SPIA	SISPA	SPIA	SISPA	SPIA	SISPA
Ct ≤ 35 (Group I)	73–8911	0.42–9.05	3.66–5.41	2.0E-3–4.3E-6	0–1.9E-2	755–7,023	0–6,123	10-GT; 1-SPP	6-GT; 3-SPP; 1-Und
								(100%; N = 11)	(90%; N = 10)
Ct ≥ 35 and ≤ 40 (Group II)	1.2–30	0.13–26.10	2.84–4.79	0–8.3E-7	0–2.5E-6	0–3,024	0–3,309	6-SPP; 3-Und.	4-SPP; 2-Und
								(67%; N = 9)	(67%; N = 6)
Ct ≥ 40 (Group III)	0.8 or less	4.68–14.95	3.99–4.75	2.6E-6 or less	0–1.1E-6	52–808	0–219	1-SPP; 3-Und	3-SPP; 1-Und
								(25%; N = 4)	(75%; N = 4)

^*^Cps total copy numbers of HuNoV input for WGS

^**^HuNoV Human norovirus, *GT* genotype NoV GII.4[P4], *SPP* species, *Und*. Undetectable, *N* sample number, *Confirmation rate* Percentage of RT-qPCR-positive samples confirmed by WGS

##### Results of SISPA-WGS on Frozen Strawberries Spiked with HuNoV

SISPA-WGS was also performed on frozen strawberry samples spiked with HuNoV. Overall, 20 RT-qPCR-positive samples from 4 sets were sequenced and 16 (80%) of these were confirmed by this strategy. Ten samples in Group I had total reads ranging from 3.66 to 5.41 M, and the percentage of HuNoV reads was 1.93 × 10^–2^ or below. With the recovered HuNoV sequences (0–6,123 bp in length), 6 out of these 10 samples were detected and identified as HuNoV GII.6[P7] at the genotype level, 3 at the norovirus species level, and 1 as undetectable (Table 4 and Supplementary Table 1 h). For the 6 samples in Group II, the percentage of HuNoV reads was at 2.52 × 10^–6^ or below. Four out of these, 6 samples were identified as HuNoV at the species level. No norovirus could be detected in the other 2 samples. Four samples in Group III obtained the percentage of HuNoV reads at 1.22 × 10^–6^ or less, from which 241 bp or fewer HuNoV sequences were recovered. Three samples were detected as containing HuNoV and in one, no virus was detected.

### WGS Analysis of Type 3 Samples: HuNoV from Naturally Contaminated Frozen Blackberries

#### Repeating RT-qPCR Analysis of the Frozen Blackberry Sample

RT-qPCR was repeated with each sampling of the frozen blackberries and no HuNoV positives were detected. The blackberry concentrates from each bag were then combined and concentrated followed by RNA isolation. The results of RT-qPCR on these concentrated samples showed that one out of three PCR replicates were HuNoV GII positive with a Ct value at 41.95 for bag A-conc., one out of three PCR replicates was positive with a Ct value at 42.65 for bag B-conc., and all of the RNA from the other aliquots and bag C-conc. were negative for HuNoV (Table 5).

**Table 5:** RT-qPCR and Sequencing results of a frozen blackberry sample naturally contaminated with human norovirus

Sample		RT-qPCR				SPIA-WGS				SISPA-WGS			
sample ID	Sub-portion	Ct ^a^	Cps ^b^	IAC Avg Cт	Positive Rep ^c^	Total reads	HuNoV reads (%)	Length (bp)	GT ^d^	Total reads	HuNoV reads (%)	Length (bp)	GT ^d^
Bag A	A-1	UD		31.10	0/3	didn't run WGS							
	A-2	UD		30.75	0/3								
	A-3	UD		32.55	0/3								
	A-4	UD		30.81	0/3								
	A-conc	41.95 or UD	34.0	39.25	1/3	3.9 M	80 (2.0E-5)	478	GII	10.4 M	0	0	-
Bag B	B-1	UD		30.86	0/3	didn't run WGS							
	B-2	UD		31.37	0/3								
	B-3	UD		30.96	0/3								
	B-4	UD		32.53	0/3								
	B-conc	42.65 or UD	21.0	36.11	1/3	4.4 M	83 (1.8E-5)	390	UA ^e^	11.2 M	40 (3.6E-6)	113	UA ^e^
Bag C	C-1	UD		31.10	0/3	didn't run WGS							
	C-2	UD		31.04	0/3								
	C-3	UD		32.69	0/3								
	C-4	UD		32.09	0/3								
	C-conc	UD		34.58	0/3								

^a^ *Ct UD* undetermined

^b^ *Cps* total copy number of norovirus recovered from 50 g blackberries, calculated based on the ratio of total volume of berry extract and the volume of extract used for RNA isolation

^c^ *Positive Rep* positive replicates of RT-qPCR assay

^d^ *GT* genotype

^e^ *UA* unassigned

#### Confirmatory Testing with WGS on the Frozen Blackberry Sample

The RT-qPCR positive RNAs from A-conc. and B-conc. were further tested/confirmed with WGS. Using the SPIA-WGS strategy, we generated 3.9 M total reads from A-conc. and 2.0 × 10^–5^% of these reads were HuNoV (Table 5). These recovered HuNoV sequences were 478 bp in length and could be identified as GII. Total reads from the B-conc. were 4.4 M total reads, of which 1.8 × 10^–5^% were from HuNoV. However, the 390 bp sequence obtained from B-conc. could not be assigned a genotype. Using the SISPA-WGS strategy, 10.4 M total reads were generated from A-conc. and 11.2 M from B-conc. There were no HuNoV reads obtained from A-conc. and the 113 bp of HuNoV sequence obtained from B-conc. could not be assigned by genotyping.

## Discussion

RT-qPCR is currently a technique employed by the FDA to detect the presence of HAV and HuNoV in foods. A positive RT-qPCR result is then followed by Sanger-based sequencing for confirmation of virus presence and virus genotyping. With the advent, advances, and popularity of whole-genome sequencing we wanted to investigate its utility as a method to confirm RT-qPCR positives and for use in genotyping the virus, particularly at very low levels of contamination.

Sequencing technologies have been applied on foodborne virus studies by many groups using various approaches and different sequencing platforms (Yang et al., 2017) (Chen et al., 2019) (Raymond et al., 2022) (Buytaers et al., 2022) (Aw et al., 2016). Bartsch et al*.* applied a metagenomics approach on frozen strawberries involved in a norovirus outbreak using the Illumina HiSeq platform (Bartsch et al., 2018). They could obtain only 2 out of 29 million sequencing reads that matched to the norovirus sequence, mainly due to the presence of highly abundant nucleic acids of other sources. Aw et al*.* could obtain rotavirus and picobirnavirus sequences from field-harvest and retail lettuce samples after sequence-independent amplification on those samples (Aw et al., 2016). Buytaers et al*.* performed sequencing using Oxford Nanopore technologies on norovirus-spiked raspberries (Buytaers et al., 2022). They showed that a norovirus genome could be obtained with shotgun metagenomics if virus is present in a sufficiently high contamination load, and with hybrid capture in lower contamination loads. These studies showed the possibilities of applying sequencing technologies to foodborne virus investigation and also demonstrated that the enrichment of viral targets, either by specific capture strategies or pre-amplification methods, could increase the virus sequencing reads and thus improve the sequencing ability on viruses in food samples. However, few studies applied sequencing on food samples containing virus in very low amounts (e.g. Ct values close to or around 40), which are the most frequently reported for viral contaminated food items.

SISPA and SPIA are two sequence-independent pre-amplification approaches that are frequently coupled with high throughput sequencing to generate viral reads from various samples (Kapusinszky et al., 2017) (Chen et al., 2018) (Chen et al., 2019) (Blomstrom et al., 2010). Myrmel et al*.* compared the efficiency of these two amplification methods combined with sequencing to recover bovine coronavirus genome (BCoV) and bovine rhinitis virus (BRBV) from nostril specimens (Blomstrom et al., 2010). Their data showed that the SPIA approach generated a higher number and a higher percentage of viral reads for both high copy number of BCoV input (4.1 × 10^5^ genome copies) and low copy number of BRBV (700 genome copies), which indicated a high efficiency of SPIA for amplification of viral RNA in comparison with SISPA. We reasoned that using pre-amplification prior to WGS for viral contaminated food samples would increase the sensitivity and improve the WGS ability to confirm RT-qPCR positive at low viral contamination levels. To this end, we used either a SPIA or a SISPA pre-amplification method prior to sequencing of HAV and HuNoV from berry samples. A serial dilution of virus ranging from 10^5^ to 10^–1^ genome copies was used to ensure coverage of low viral quantities. Our data showed that either SPIA or SISPA coupled with WGS could recover enough reads of HAV or HuNoV from samples in Group I (Ct ≤ 35) for confirmation and genotyping. For samples in Group II (Ct ≥ 35 and ≤ 40) or III (Ct ≥ 40), which had lower amounts of virus input, they both could confirm some but not all RT-qPCR-positive HAV and HuNoV samples. In addition, due to the limited number of sample replicates (especially in Group III), a comparison of efficiency, as well as the limit of detection of SPIA-WGS and SISPA-WGS for confirmation of HAV and HuNoV, was not performed. Studies specifically designed to determine the limit of detection for confirmation and genotyping are warranted.

Three types of berry samples containing either HuNoV or HAV were included in this study. For Type 1 samples, HAV- or HuNoV RNA transcripts were directly added to raspberry RNA extracts and provided an ideal model to examine the virus detection by WGS. This model allowed us to use a pre-determined number of viral RNA copies for RT-qPCR and WGS without the need to consider virus recovery yield, intact virus, and viral genome integrity. Our data showed that the positivity of HAV transcripts could be consistently confirmed using either SPIA-WGS or SISPA-WGS when the Ct values were less than 40.

When the Ct values were above 40, 9 out of 13 (69%) PCR-positive samples could be confirmed by SPIA-WGS, while 9 out of 9 (100%) were confirmed by SISPA-WGS (Table 1). Notably, both pre-amplification-WGS strategies were able to detect the presence of 0.1cp/3 µL viral RNA in samples (4 out 5 samples and 1 out of 2 samples for SPIA and SISPA, respectively) that had previously tested negative by RT-qPCR (Supplementary Table 1a and 1b). This might be due to a lack of viral RNA transcript in the 3 µL of sample used in the RT-qPCR reaction. In the case of frozen raspberry samples spiked with HuNoV transcripts (Type 1), all 20 PCR-positive samples could be confirmed by SPIA-WGS. However, only 3 out of 5 (Ct between 35 and 40) and 2 out of 5 (Ct > 40) samples were detected by SISPA-WGS, suggesting that SPIA-WGS might provide better performance for detecting HuNoV transcripts in samples with higher Ct values.

The Type 2 samples contained viral RNA derived from either a HuNoV-positive stool sample (a natural model of virus contamination) or HAV virus from cell culture spiked onto frozen strawberries. In these samples, HAV and HuNoV at low levels (Ct values close to 40) could be detected by both SISPA-WGS (Supplementary Table 1f and 1 h) and SPIA-WGS (Table 3). However, a 1:10,000 HAV dilution (Supplemental Table 1f Spiking 2) was identified at the genotype level by SISPA-WGS but was not detected by SPIA-WGS (Table 3). For the HuNoV-spiked samples (Table 4), 18 out of 24 and 16 out 20 PCR-positive samples could be confirmed with SPIA-WGS and SISPA-WGS, respectively. For the samples with Ct values higher than 40, 1 out 4 could be confirmed by SPIA-WGS, 3 out 4 could be confirmed by SISPA-WGS. With the limited number of samples, it is hard to demonstrate if SISPA-WGS had better performance than SPIA-WGS on confirmation of human norovirus samples with higher Ct values.

For the naturally contaminated blackberry (Type 3) sample, two out of three bag samples were determined as GII positive at high Ct values (46.5 and 43.4) prior to our receipt of the samples. Despite using the same isolation/detection protocol (the BAM protocol), we could not repeat/achieve positive results for any of the 12 × 50 g samplings from the 3 bags. This could be attributed to three possibilities: first, virus contamination is unevenly distributed in the samples; second, viral RNA was absent in the 3 µL volume used for the PCR reactions due to its low concentration; or third, the original RT-qPCR results were false positives. To address these possibilities, the remaining four berry concentrates derived from the same bag for each of the three bags were combined and concentrated prior to RNA isolation. RT-qPCR results showed that 2 out of 3 bags were HuNoV GII positive with pooling and concentration of the concentrates, although only one out of three PCR replicates was positive with a Ct of 41.95 and 42.65 for bag A and B, respectively (Table 5). These two PCR-positive RNA samples were subsequently used for sequencing. HuNoV reads from bag A were recovered and assigned as HuNoV GII by SPIA-WGS but not SISPA-WGS, while HuNoV reads from bag B were unassigned by both SPIA-WGS and SISPA-WGS.

In contrast to the strawberry extract spiked with HAV transcripts, from which nearly full-length HAV genomic sequences still could be recovered for some of the samples with Ct above 40, only partial viral genomic sequences could be generated for most of the HuNoV transcripts-spiked samples having Ct above 30; this was also true for all HAV or HuNoV-spiked strawberries by WGS. Our data also showed that a larger percentage of target viral reads and longer viral sequences were obtained with more virus input and very few reads were obtained from samples with high Ct (e.g. close to or above 40). Thus, if the recovered partial viral sequences were from regions outside of the genotyping location, the samples could be detected at species level but not identified at genotype level.

Our data show that extremely low levels of viral RNA in samples that were negative according to RT-qPCR could sometimes be detected using WGS techniques. We took steps to ensure that we could discriminate a true positive from cross contamination, including performing all the steps for sample preparation and RNA work in separate areas, running a negative control for both RT-qPCR and WGS assays, and taking additional precautions to avoid cross contamination during sequencing, such as stringent washing of the sequencer with Tween 20 between runs, and stringent QC to remove reads with low-quality scores and aligning the recovered sequences with the sequence database from in-house samples to exclude any cross contamination.

Our results indicate that RNA concentration could be one of the options to improve the capability of the current BAM detection method. Similar results were also observed with the HuNoV-spiked strawberries (spiking 4, Supplementary Table 1 g and 1 h). Instead of concentrating berry concentrates as above, isolated virus RNA was combined and concentrated for the RT-qPCR assay. Ct values dropped from 34.6 and 36.4 to 32.1 for the samples with the spiking at concentration of 1:1000 dilution. Similarly, Ct values dropped from 37.2 and 38.8 to 35.2 for spiking at 1:10,000 dilution. These concentrated RNA samples were detected by both SPIA-WGS and SISPA-WGS with higher percentage of HuNoV reads in comparison with its unconcentrated counterpart (spiking 4, Supplementary Table 1 g).

Data from the concentrated RNA samples showed both an improved sensitivity of RT-qPCR and an increase of WGS viral reads. Thus, it may be useful to consider how to optimize the protocols by adding a concentration step to improve the sensitivity of existing detection and confirmation methods.

Lastly, application of WGS methods for foodborne virus detection will not only provide a confirmatory method on RT-qPCR results but also has other potential advantages over RT-qPCR in certain contexts as shown in our results. Specifically, in comparison with RT-qPCR targeting only a short genomic fragment, longer viral genome sequences are usually recovered with WGS, indicating a large part of the viral genome is present in the sample. It is possible to recover the virus fragments with WGS, even from the regions out of the RT-qPCR targeting, thus catching the “positive” when the signals are missed by RT-qPCR in the targeted short region. In addition, with the sequences obtained by WGS, the virus could be directly genotyped without other assays. Such information could be valuable for interpreting “positive” RT-qPCR results by identifying the virus strain and thus aiding in the tracking of the contamination source(s) in outbreaks.

## Conclusion

Accurate detection of foodborne viruses and reliable confirmation of those results are essential for outbreak investigations, as well as for preventive surveillance studies. In this study, our non-targeted WGS strategy following pre-amplification with random primers was used to confirm positive RT-qPCR results. Results from three different berry sample models containing either HAV or HuNoV (RNA transcripts, spiked viruses, and naturally contaminated blackberry samples) demonstrated that our non-targeted WGS using either SPIA or SISPA pre-amplification could confirm viruses at a very low level. However, the low number of viral reads relative to the high number of total sequencing reads suggests further method improvement and standardization are needed before routine application. Nonetheless, WGS approaches are promising and could be potentially developed as confirmatory methods for viral detection and outbreak investigation. Further method optimization research, including removing background nucleotides, concentrating the samples, and/or using specific virus-targeted amplification, may improve the results.

### Supplementary Information

Below is the link to the electronic supplementary material.

Supplementary file1 (XLSX 59 kb)

## Data Availability

Data will be made available on request.
